# Relationships between Categorical Perception of Phonemes, Phoneme Awareness, and Visual Attention Span in Developmental Dyslexia

**DOI:** 10.1371/journal.pone.0151015

**Published:** 2016-03-07

**Authors:** Rachel Zoubrinetzky, Gregory Collet, Willy Serniclaes, Marie-Ange Nguyen-Morel, Sylviane Valdois

**Affiliations:** 1 Centre Référent des Troubles du Langage et des Apprentissages, Pôle Couple-Enfant, Centre Hospitalier Universitaire, Grenoble, France; 2 Unité de Recherche en Neurosciences Cognitives, Centre de Recherches en Cognition et Neurosciences, Université Libre de Bruxelles, Brussels, Belgium; 3 Laboratoire Psychologie de la Perception, CNRS, UMR 8242, and Université Paris-Descartes, Paris, France; 4 Laboratoire de Psychologie et NeuroCognition, CNRS, UMR 5105, F-38000, Grenoble, France; 5 Université Grenoble Alpes, LPNC, CS 40700, F-38058, Grenoble, France; University of Copenhagen, DENMARK

## Abstract

We tested the hypothesis that the categorical perception deficit of speech sounds in developmental dyslexia is related to phoneme awareness skills, whereas a visual attention (VA) span deficit constitutes an independent deficit. Phoneme awareness tasks, VA span tasks and categorical perception tasks of phoneme identification and discrimination using a d/t voicing continuum were administered to 63 dyslexic children and 63 control children matched on chronological age. Results showed significant differences in categorical perception between the dyslexic and control children. Significant correlations were found between categorical perception skills, phoneme awareness and reading. Although VA span correlated with reading, no significant correlations were found between either categorical perception or phoneme awareness and VA span. Mediation analyses performed on the whole dyslexic sample suggested that the effect of categorical perception on reading might be mediated by phoneme awareness. This relationship was independent of the participants’ VA span abilities. Two groups of dyslexic children with a single phoneme awareness or a single VA span deficit were then identified. The phonologically impaired group showed lower categorical perception skills than the control group but categorical perception was similar in the VA span impaired dyslexic and control children. The overall findings suggest that the link between categorical perception, phoneme awareness and reading is independent from VA span skills. These findings provide new insights on the heterogeneity of developmental dyslexia. They suggest that phonological processes and VA span independently affect reading acquisition.

## Introduction

Many different theories have been proposed to account for developmental dyslexia (DD), including the phonological theory [1, 2] and several visual or visual-attentional theories [3–6]. The phonological and visual magnocellular theories, initially considered as concurrent, are now more likely viewed as related since the magnocellular dysfunction typically co-occurs with the phonological disorder [7–9]. In the same way, sluggish attentional shifting [5] and attention orienting disorders [10] typically co-occur with phonological disorders in DD [11–14]. By contrast, the visual attention (VA) span disorder, defined as reduced multi-element simultaneous processing [3], is typically found in children who have no phonological problem [15–18], thus suggesting that VA span and phonological abilities may be two independent cognitive underpinnings of DD [3, 19, 20].

Besides, low-level perceptual deficits have been studied as a potential cause of the phonological disorder in DD. Children with dyslexia have been reported to have poor categorical perception (CP) of speech sounds, which could affect their phonological processing skills and hamper the set-up of grapheme-phoneme mappings (see [21] for a recent meta-analysis). This theoretical framework assumes that CP should relate to phonological skills. Assuming that phonological skills and VA span abilities are independent cognitive deficits in DD, we should expect no relationship between VA span and CP abilities. The current study aims at providing additional support for a relationship between phonological skills and CP in children with DD. For the first time, we will explore whether this relationship is specific and distinct from the VA span disorder.

### CP in developmental dyslexia

The most consensual cognitive deficit in DD is a phonological awareness deficit (see [22] for a review and meta-analysis). The potential causes of this phonological deficit have been further investigated and different types of auditory sensory dysfunctions have been reported [23]. Impairments in the ability to process the acoustic structure of speech sounds should affect phonological processing and thus appear as a potential cause of the phonological disorder in DD. In line with this expectation, a speech perception deficit has been evidenced in DD, most often through syllable discrimination tasks: dyslexic children are less efficient to discriminate pairs of consonant-vowel (CV) syllables that differ on a single phonological feature, as place of articulation (e.g. between /ba/ and /da/), or voicing (e.g. /ta/ and /da/) [24–27].

Phoneme discrimination reflects CP abilities, i.e. the ability to perceive differences between phonemes while ignoring acoustic differences between the variants of the same phoneme [28]. CP can be assessed by collecting identification and discrimination responses to stimuli varying along some acoustic continuum. The identification task reveals how efficiently listeners can attach phonemic labels to the acoustic stimuli. The discrimination task measures their ability to judge two acoustic segments of the continuum as similar or different. A large array of studies has shown that dyslexic individuals have a weaker degree of CP. They show weaker accuracy in discriminating acoustic differences across phonemic boundaries but enhanced discrimination of acoustic differences within the same phoneme category, i.e. an enhanced discrimination of allophonic differences (see [21] for a meta-analysis, [29–34]). Enhanced discrimination skills for intra-categorical stimuli suggest an ‘allophonic’ mode of perception in DD, i.e., the allophonic variants of the same phoneme are analyzed as distinct phonemes.

The perception of acoustic features is universal, perceptually invariant and language independent. Accordingly, the perception of universal features emerges quite spontaneously after a few months of exposition to language. In contrast, phonemes are specific to each language and the acquisition of language-specific phonemic boundaries requires combining the universal psychoacoustic boundaries in a specific way. This acquisition is delayed during perceptual learning and typically occurs after at least six months of language exposure. However, some dyslexic childdren may not restrict their acoustic analysis to language specific boundaries. They remain sensitive to differences between sounds that belong to different phonemic categories in other languages but are not relevant for their own language, thus showing an allophonic mode of perception. For illustring this point, take the example of the voicing opposition. Differences in voice onset time (VOT) are used to discriminate between voiced and voiceless phonemes (for voiced phonemes: VOT is negative because the onset of vocal vibrations begins before the burst of the plosive consonant; for voiceless phonemes: VOT is positive because the onset of vocal vibrations begins after the release of the plosive). During early childhood, before around six months of age, the child is sensitive to the universal allophonic VOT boundaries that are located at some -30 ms and +30 ms VOT. These phonemic boundaries remain relevant in some languages but in other languages as French, the voicing boundary is located at 0 ms VOT. The acquisition of the VOT phonemic boundary in French derives from a coupling between the allophonic boundaries [35, 36]. A dyslexic child who would be sensitive to the allophonic (+30 ms or -30 ms) VOT, would show an allophonic perception. Different sources of evidence, both behavioral and neural, suggest that increased sensitivity to intra-categorical variants of the same phoneme in children with DD is due to the persistent use of allophonic features for perceiving the phonemes of their native language (see [37] for a review).

To summarize, the CP deficit in dyslexia is charaterized by both reduced sensitivity to acoustic differences across phonemic boundaries (i.e., deficit in phonemic precision) and enhanced sensitivity to differences within phonemic categories (i.e., allophonic perception). The latter is in most instances evidenced by the discrimination data but not by the identification data, which gives rise to a deficit in “classical” CP, i.e. to a weaker relationship between discrimination and identification responses [28].

Both the deficits in phonemic precision and allophonic perception affect the robusteness of phonemic representations which might in turn affect reading acquisition. The implications of allophonic perception are straightforward. As it blurs the relationships between letters and speech units, allophonic perception is a possible cause of DD whatever the language transparency. Indeed even in a perfectly transparent orthographic system, allophonic sensitivity should lead to associate several different allophones to each grapheme, thus making the grapheme-phoneme relationships more confused and complex.

### CP and Phonological skills

The CP deficit was not consistently reported in DD. While some studies showed clear evidence for a CP disorder, the deficit was sometimes marginally significant [31], or not found at all in other studies (see [38] for a review and meta-analysis on 50 studies). Such discrepancies can be attributed to methodological differences (e.g., as the use of different tasks) [21] but they could also reflect heterogeneity in the dyslexic population. Indeed, the phoneme discrimination deficit has sometimes been found to characterize just a subgroup of dyslexic individuals [24, 26, 39–42]. Manis et al. [39] used an identification task on a /p/—/b/ continuum of VOT to estimate CP in two subgroups of dyslexic children with or without phoneme awareness disorders. The authors reported a CP disorder but only in the subgroup of dyslexic children with poor phoneme awareness. They further found that CP and phoneme awareness performance correlated moderately but significantly in the whole sample of dyslexic participants, chronological age and reading age matched controls. However when the population was restricted to dyslexic children, some studies reported significant correlations between phoneme identification and phoneme awareness [43] but others did not [40]. Investigation of cortical responses to sound discrimination in DD also failed to reveal atypical functioning in children with phonological disorders [42]. By contrast, studies on typical readers provided evidence for a link between CP and phoneme awareness [44, 45]. McBride-Chang [46] further explored the relationships between CP, phoneme awareness and word reading performance. She showed that the relationship between phoneme identification and reading was mediated by phoneme awareness in typically developing children.

Recent findings from a remediation study in children with Specific Language Impairment (SLI) further suggest that CP may causally relate to phoneme awareness [47]. Children with SLI benefited from a two-weeks phoneme-discrimination training program. After training, they showed sharper categorical boundaries than a matched control group of untrained SLI children. Critically, discrimination training not only improved their CP but further their phoneme awareness skills.

To summarize, there is consistent evidence for a relationship between CP and phoneme awareness in typical children. By contrast, we lack strong evidence that the CP deficit relates to poor phoneme awareness in DD and/or that impaired CP mainly characterizes a subgroup of children with poor phonological skills. Discrepant results across studies may result from the heterogeneity of the dyslexic population and the difficulty to identify cognitively-homogeneous subgroups of dyslexic children.

### Cognitive heterogeneity in the dyslexic population

Most studies on CP in DD have been carried out on unselected groups of dyslexic children without consideration for their potential cognitive heterogeneity. The few studies that tried to identify a phonologically impaired subgroup used various criteria (poor phoneme awareness or poor decoding skills) and did not systematically control for additional comorbid deficits as SLI [40] or attention deficit hyperactivity disorder (ADHD) [43] that seem to affect CP and its relationship with phoneme awareness. Furthermore, the phonologically impaired subgroup when identified was compared to cognitively unspecified subgroups of dyslexic children (mainly defined by exclusion criteria), which might have decreased the probability to find reliable evidence for a specific relation between CP and phonological disorders. Our aim in the current study was to better characterize our dyslexic population through assessment of phonological and VA span abilities.

VA span abilities correspond to the number of distinct visual elements that can be processed simultaneously, regardless of their verbal or non verbal nature [48]. Case studies showed that some dyslexic individuals exhibited reduced VA span abilities [15, 17, 18] and that VA span and phonological disorders could dissociate in developmental dyslexia [11, 16]. Large scale studies revealed the existence of a subset of dyslexic children who exhibited a VA span disorder but preserved phoneme awareness while another subset showed poor phoneme awareness but preserved VA span [3, 19, 20]. These studies further revealed that the VA span disorder contributed to the poor reading outcome of dyslexic children, independently of their phoneme awareness skills. Studies carried out on typically developing children provided support for the independent contribution of VA span and phonological skills to reading performance [49] and suggested special links between VA span and reading speed [48, 50].

Neuro-imaging investigations identified the superior parietal lobules as the neuronal underpinnings of the VA span [51–54]. Atypical activity of such superior parietal regions in dyslexic individuals with a VA span disorder contrasts with previous reports of a left perisylvian dysfunction in DD. To explore more in depth whether different cognitive deficits might relate to different neuronal underpinnings, Peyrin et al. [55] explored the brain activity of two young adults with DD who were engaged in two phonological and visual categorization tasks under fMRI. The two participants who were matched for their reading level nevertheless showed dissociated cognitive deficits, either a phonological or a VA span deficit. Critically, under-activation of the perisylvian regions of the left hemisphere was found in the participant with a single phonological disorder but not in the VA span-impaired participant. Reversely, the participant with a single VA span disorder showed under-activation of the superior parietal lobules, the activation of which was preserved in the phonologically impaired participants. The whole findings strongly suggest the existence of different subtypes of DD characterized by distinct cognitive disorders and distinct neurobiological dysfunctions.

In the current paper, we will investigate whether previously reported discrepant results for or against a CP deficit in DD may follow from the cognitive heterogeneity of the dyslexic population. We will explore whether the relationship previously reported between CP and phoneme awareness in typical readers [39] extends to DD and whether the CP deficit might be specific to the subgroup of dyslexic children with a phoneme awareness disorder. In a first section carried out on the whole dyslexic sample, we will compare the CP performance of dyslexic children to that of chronological age matched controls. We will then focus on the dyslexic population and explore the relations between the key variables—CP, phoneme awareness, VA span, and reading (speed and accuracy)—through correlation and mediation analyses. In the second section, we will identify two cognitively distinct subgroups of DD defined by either a single phoneme awareness or a single VA span deficit and assess whether the phonological group alone exhibits a CP deficit, i.e., significantly lower CP than the controls.

## Part 1. Relationship between CP, Phoneme Awareness and VA Span in the Whole Dyslexic Sample

In this first part of the study, we will compare children with dyslexia with chronological-age-matched controls to replicate previous evidence of allophonic perception, and the related CP deficit in the dyslexic population. Using a VOT continuum, we postulated that dyslexic children would be less sensitive than controls to differences across the phonemic boundary, that is located around 0 ms VOT in French, and that these children would be more sensitive than controls to differences across allophonic boundaries, that are located at -30 and +30 ms VOT. These hypotheses will be tested through tasks of phoneme discrimination and identification. Given that allophonic sensitivity is more readily assessed using discrimination (but not identification) data, the identification scores will be converted in “predicted” discrimination scores following the classical procedure described in the literature [56].

Correlation analyses will also be computed, in search for positive correlations between phoneme awareness and CP. We will further explore how these two processes relate to VA span abilities and reading skills. While phoneme awareness is expected to correlate with CP, the latter should not relate to VA span. Besides as largely documented in the literature, we should replicate positive relationships between phoneme awareness and reading accuracy and will further explore whether CP correlates with reading performance. We will also perform mediation analyses to assess whether the link between CP and reading accuracy is mediated by phoneme awareness.

### Method

#### Participants

Sixty-three children with dyslexia (mean age = 10 years 6 months, standard deviation (SD) = 15 months) and 63 control children (mean age = 10 years and 1 months, SD = 11 months; F(1,124) = 3.3, p = .071) participated in this study. All were French native speakers who had normal hearing and normal or corrected-to-normal vision. They attended school regularly and none of them had any history of neurological illness or brain damage. All the participants and their parents gave their written consent to participate to the study. The local Grenoble Ethics committee approved the study.

The typically developing children were recruited from schools of the Grenoble urban area. They reported no history of oral language or reading disorder and showed a normal reading age on the Alouette Reading Test [57] (mean reading age = 10 years and 10 months, SD = 19 months).

The dyslexic children were recruited at the center for learning disabilities of the Grenoble University Hospital or in speech therapy offices where they received a complete medical and neuropsychological assessment. All had a normal IQ (exclusion if score<25e percentile on the Raven’s Progressive Matrices [58] or if Verbal Comprehension Index and Perceptual Organization Index < 85 on the Wechsler Intelligence Scale for Children—WISC IV [59]). Children with associated SLI or ADHD were not included. The dyslexic participants’ reading age was significantly lower than for the control group (mean reading age = 7 years and 5 months, SD = 8 months; F(1,124) = 236.5, p < .001).

#### Cognitive assessment

The dyslexic children were administered three reading tests of isolated regular and irregular word and pseudo-word reading, three phoneme awareness tasks of phoneme deletion, phoneme segmentation and acronyms, and two tasks of global and partial report to assess their VA span abilities. A control task of single letter processing was further administered. The tasks were presented in a random order that varied from one child to the other. The children were tested individually in one or two sessions for a total assessment duration of approximately two hours interrupted by a break (additional breaks could also be requested by the child at any time).

***Reading tasks*:** For the reading assessment, the participants were asked to read aloud the 80 words (40 regular and 40 irregular) and 40 pseudo-words of the ODEDYS neuropsychological battery [60]. The regular and irregular word lists were matched for letter and syllable length, grammatical class and frequency. The 40 pseudo-words were legal pseudo-words without lexical neighbors. Both accuracy and reading speed were taken into account.

***Phoneme awareness tasks*:** Phoneme awareness was assessed using the tasks of phoneme deletion and phoneme segmentation from Bosse & Valdois [49], and the acronyms task from the BELEC battery [61]. For each task, the participants were given a set of practice items for which they received feedback. No feedback was provided on the experimental items. We recorded the percentage of correct responses. In the phoneme deletion task, the participants had to delete the first sound of a spoken word and produce the resulting pseudo-word (e.g., “outil” /uti/: /ti/; “placard” /plakaR/: /lakaR/). Twenty experimental words were presented. Seven words began with a vocalic phoneme corresponding to a multiple letter grapheme so that the omission of the first letter (instead of the first phoneme) yielded incorrect responses, 9 began with a consonantal cluster, 4 with a singleton. In the phoneme segmentation task the participants had to successively sound out each phoneme of a spoken word (e.g. /kado/ ‘cadeau’ gift: /k/- /a/- /də/- /o/). Fifteen words made up of 4 phonemes on average (from 3 to 5) were presented. In the acronyms task, two oral words were successively presented. The children had to extract the first phoneme of each word and blend them to produce a new syllable (e.g. “photo” “artistique” /foto/-/aRtistik/ says /fa/). The test comprised 10 series of two words made up of 4.4 phonemes on average (range 2–8). Seven words began with a phoneme corresponding to a digraph so that children generated an erroneous word if the first letter was extracted instead of the first phoneme (response /pa/ instead of /fa/ if orthographically biased in the above example).

***VA span tasks*:** The global and partial letter report tasks were administered to assess VA span abilities. A task of single letter identification threshold was further administered to control for single letter processing. The tasks were displayed on a PC computer using E-prime software (E-prime Psychology Software Tools Inc., Pittsburgh, USA). The strings were made of black upper case (Arial, 7 millimeters high) letters displayed on a white background at the center of the screen.

*The tasks of global and partial report*: Stimuli were random five letter-strings (e.g., RHSDM; angular size = 5,4°) built up from 10 consonants (B, P, T, F, L, M, D, S, R, H). The consonant strings contained no repeated letters and did not match the skeleton of a real word (e.g.: FLMBR for FLAMBER “burn”). Two subsequent letters never corresponded to a French grapheme (e.g. PH, TH) or a frequent bigram in French (e.g. TR, PL, BR). The distance between adjacent letters was of 0.57° in order to minimize crowding. Twenty 5-letter strings were displayed in Global Report. Each letter was used ten times and appeared twice in each position. Fifty random 5-letter strings were used in Partial Report. Each letter occurred 25 times (5 times in each position). At the beginning of each trial, a central fixation point was presented for 1000 ms followed by a blank screen for 50 ms. Then, a horizontal 5-letter-string was displayed centered on fixation for 200 ms, a duration which corresponds to the mean duration of fixations in reading, long enough for an extended glimpse, yet too short for a useful eye movement. In the Global report condition, children had to report verbally all the letters they had seen immediately after the string disappeared. In Partial Report, a vertical bar cueing the letter to be reported was displayed 1.1° below the target letter, at the offset of the letter-string. Each letter was used as target once in each position. Participants were asked to report the cued letter only. In both tasks, the experimenter pressed a button to start the next trial after the participant’s oral response. The experimental trials were preceded of 10 training trials for which participants received feedback. No feedback was given during the experimental trials. Score was the number of accurately reported letters across the 20 trials in Global report (regardless of order; maximum score: 100) or across the 50 trials in Partial report (maximum score: 50).

*The single letter identification task*: The task was designed to control for single letter processing skills. Each of the 10 letters used in the report tasks were randomly presented (5 times each) with the same physical characteristics as in the experimental tasks, at 5 different presentation durations (33, 50, 67, 84 and 101ms). At the offset of the letter, a mask (13 mm high, 37 mm wide) was displayed for 150 ms. Participants were asked to name each letter immediately after its presentation. The test trials were preceded of 10 practice trials (2 for each presentation time) for which participants received feedback. Children were excluded when the maximal score of 10 accurate identifications was not reached at one of the presentation durations. The total score was the sum of scores at each of the display durations.

#### Categorical perception tasks

A /də/-/tə/ VOT continuum, from -75 to +75 ms VOT in 30 ms step, was synthetized by a parallel formant synthesizer provided by Carré [62]. VOT is negative when the onset of vocal vibrations begins before the burst of the plosive, VOT is positive when the onset of vocal vibrations begin after the release of the plosive. F1, F2 and F3 transitions frequencies were 200, 2200 and 3100 Hz, respectively, and the steady-state formant parts were 500, 1500 and 2500 Hz, respectively. F0 frequency was maintained constant at 120 Hz. Each syllable of the continuum was 200 ms long. Previous studies that used the same stimuli have shown that both French-speaking typical children [63] and French-speaking SLI children [47] perceived negative VOT stimuli as /də/ and positive VOT stimuli as /tə/.

The identification and discrimination tasks were administered to the dyslexic and control participants. In order to facilitate the association between sounds’ perception and the collection of answers, children were introduced to two different cartoons from a children’s book (named Dom and Tom). Each cartoon was associated with a specific syllable (/də/ or /tə/ respectively). The stimuli were binaurally delivered through headphones (Sennheiser HD 202).

*The identification task*: Prior to testing, the participants completed a familiarization task composed of one block of twenty stimuli (ten trials of each VOT endpoints values of the continuum: -75 ms and +75 ms VOT) presented in a random order that differed for each session and each participant. The children had to associate each presented sound with a dedicated cartoon by pressing on the keyboard the “1” key if they heard the syllable /də/ and the “0” key if they heard the syllable /tə/. To facilitate this association, the cartoons were displayed at the bottom of the screen, Dom being located on the left (near the “1” key) and Tom on the right (near the “0” key); the keyboard was placed centrally in front of the screen. There was a 2000 ms interval between the child response and the following item. Following each answer, a feedback was provided on the screen (a red screen for incorrect answers, a picture of a gift displayed at the screen center for a correct answer). At the end of the familiarization session, the experimental identification task was presented in one block of sixty stimuli (10 trials for each of the VOT values: -75, -45, -15, +15, +45, and +75 ms VOT), which were displayed in a random order that differed for each session and each participant. No feedback was provided during the experimental task.

*The discrimination task*: Prior to testing, a familiarization task using the endpoints stimuli of the continuum was provided. One block composed of twenty pairs of sounds (5 trials of each of the following pairs: -75/-75 ms “/də/-/də/”, -75/+75 ms “/də/-/tə/”, +75/-75 ms “/tə/-/də/” and +75/+75 ms “/tə/-/tə/” VOT) was built up and presented in a random order, different for each session and each participant. For each item, two pairs of identical cartoons (Dom-Dom and Tom-Tom) were displayed on the right side of the screen close to the “0” key that had to be pressed when the two successively displayed sounds were the same, i.e. for the “/də/-/də/” and “/tə/-/tə/” pairs. The two pairs of different cartoons (Dom-Tom and Tom-Dom) were on the left side close to the “1” key that had to be pressed when the pair sounds were different. The same key was pressed whatever the order of the two successive sounds (/də/-/tə/ or /tə/-/də/). The participants had to press the response key at the offset of the sound pair. The child’s response corresponded to their judgment of the two successive sounds as identical or different, as a single key was used for the responses Dom-Dom and Tom-Tom and a single key for the Dom-Tom and Tom-Dom responses. There was a 100 ms interval between the pair’s stimuli and a 2000 ms interval between the child response and the following item. A feedback was provided on the screen (a red screen for incorrect answers, the picture of a gift at the screen center for correct answers). The discrimination task was subsequently presented to each child. It was composed of a set of eighty pairs of stimuli that were displayed in a random order that differed for each session and each participant (five trials of each of the eight identical pairs: -75/-75, -45/-45, -15/-15, +15/+15, +45/+45, and +75/+75 ms VOT; and five trials of each of the ten different pairs: -75/-45, -45/-75, -45/-15, -15/-45, -15/+15, +15/-15, +15/+45, +45/+15, +45/+75, and +75/+45 ms VOT). Neither positive nor negative feedback was provided during this task.

#### Statistical analyses

The observed discrimination curve was compared to the predicted discrimination curve derived from the identification data. Predicted discrimination was computed using elementary probability formulas [56] that were adapted to an AX discrimination paradigm (with a binary choice between /də/ and /tə/). The d’ scores were taken as the dependent variable.

Two kinds of group comparison were performed, focusing on targeted VOT or on a general measure of boundary precision. The former comparisons focused on the VOT pairs corresponding to either the universal psychoacoustic boundaries (-30 ms and +30 ms VOT) or the French-specific phonemic boundary (0 ms VOT). Discrimination differences between groups were tested using repeated-measures ANOVAs with VOT (central value of each pair, three levels: -30 ms, 0 ms, +30 ms VOT) and Task (two levels: predicted vs. observed discrimination) as within-subject factors, and Group as between-subjects factor. If dyslexic children are less sensitive than controls to differences across the phonemic boundary (0 ms VOT), but more sensitive than controls to differences across allophonic boundaries (-30 and +30 ms VOT), then both a Group by VOT interaction and a specific Group effect on the targeted VOT values should be observed. Finally, we expected to find larger differences between groups with the discrimination tasks because CP deficit is stronger, on meta-analytic grounds, for discrimination than for identification [21].

Assuming that individual differences in the location of the phonemic boundary might undermine the inferences based on the assumption that this boundary is fixed at 0 ms VOT, further analyses were conducted on the individual discrimination peaks calculated separately for each participant and for each task. A peak was then defined as the VOT pair that collected the largest discrimination score. Both the amplitude of these peaks (in d’ values) and their locations along the VOT continuum (in ms) were calculated. If the phonemic boundary perceived by the dyslexic children is less accurate regardless of peak location, then the peak amplitude should be lower for the dyslexic children than for controls when the location of the predicted peak is entered as co-variable.

Partial correlation analyses controlling for age were further computed to assess the links between the different variables. Positive and significant correlations were expected between CP measures (at the phonemic and universal boundaries, and at the highest discrimination peak), phoneme awareness and reading performance but not between the two first variables and VA span. In line with previous findings, significant links were further expected between both phoneme awareness and reading, and VA span and reading but not between phoneme awareness and VA span. As previously reported, phoneme awareness would correlate with reading accuracy more than with reading speed [64] while reversely, VA span should more specifically relate to reading speed [3, 49].

Last, a mediation analysis was performed to explore whether the relationship between CP and reading was mediated by phoneme awareness. We further expected the relationship between these variables not to be sensitive to entering VA span skills in the mediation analysis, which would suggest an independence of the CP/phonological skills and VA span.

### Results

#### Cognitive performance of the dyslexic group

Performance of the dyslexic group on the tasks of reading, phoneme awareness and VA span is presented in Table 1. Z-scores were calculated from the mean and standard deviation of populations of the same chronological age taken from the BALE neuropsychological battery [65] for the reading tasks and from Bosse & Valdois [49] for the VA span tasks. For the phonological tasks, the data collected on the current control group were used to calculate the corresponding Z-scores. The dyslexic group was characterized by a 36 months delay on average in reading acquisition, showing that the dyslexic participants exhibited a severe reading disorder. Both word (regular and irregular) and pseudo-word reading (accuracy and speed) were severely impaired, suggesting impaired development of the two, global and analytic, reading procedures. The group’s phonological skills were within the normal range, even if a subgroup of participants did exhibit a severe phonological disorder. The dyslexic group’s VA span abilities were slightly lower than for the controls (<-1 SD) but here again some children showed a clear VA span disorder while others performed within the normal range. Letter identification skills were within the normal range. The group’s cognitive heterogeneity will be explored more in depth below.

**Table 1:** Characteristics of the dyslexic group.

	Mean score	SD	Min	Max	Mean Z-score	SD
Age (months)	125,54	15,22	94	153		
Reading Age (months)	89,33	7,75	79	122		
Reading delay (months)	36,89	14,88	15	83		
RW score (/20)	15,69	3,44	3	20	-2,46	2,31
RW time (sec)	43,76	19,18	15	101	-2,66	2,20
IW score (/20)	11,21	4,43	2	20	-1,95	1,52
IW time (sec)	49,98	21,83	15	120	-2,41	2,19
PW score (/20)	12,20	3,71	4	18	-2,22	1,60
PW time (sec)	52,25	18,36	18	115	-1,95	1,66
Deletion (%)	71,11	19,52	30	100	-0,80	1,33
Segmentation (%)	59,26	25,15	7	100	-0,06	0,96
Acronym (%)	72,38	21,61	0	100	-0,48	1,10
Whole report (%)	70,76	11,58	41	94	-1,14	1,05
Partial report (%)	74,79	14,44	24	100	-1,20	1,39
Letter identification (/50)	44,65	5,09	25	50	0,24	0,67

Mean and Z-scores, standard deviations (SD) and ranges for chronological age, reading age, regular word (RW), irregular word (IW), and pseudo-word (PW) reading, and phonological and visual attention span skills for the dyslexic participants.

#### Comparison of the dyslexic and control groups on CP skills

**Fig 1:**
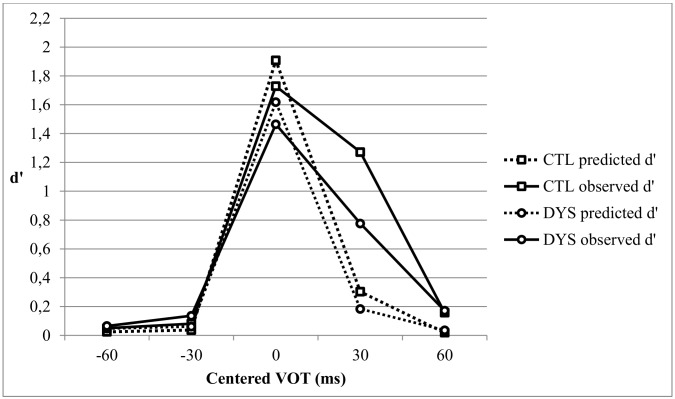
Predicted and observed discrimination curves for the Control (CTL) group and the Dyslexic (DYS) group.

Fig 1 shows the actual discrimination d’ scores (observed d’) and those predicted from the identification data (predicted d’) for the dyslexic (DYS) and control (CTL) groups.

A repeated measures ANOVA was performed with Task (Observed vs. Predicted) and VOT (3 pairs centered on -30, 0, and +30 ms VOT) as within-participant variables, and Group (DYS vs. CTL) as between-participants variable. Results showed a significant Group effect [F(1,124) = 7.65, p < .01, η^2^ = .058]. The Group x VOT interaction [F(2,248) = 2.63, p = .092, Greenhouse-Geisser corrected, η^2^ = .027], and the Task x VOT x Group interaction [F(2,248) = 2.02, p = .141, Greenhouse-Geisser corrected, η^2^ = .016] were not significant.

Further analyses were performed to assess the a priori hypothesis of between-group differences at the targeted VOT values. The Group effect was tested with repeated measures ANOVAs separately for each targeted pair with Task as within-participant variable. There were no significant differences between groups for the pair centered at -30 ms VOT (F<1) but a trend towards a Group effect [F(1,124) = 2.81, p = .096, η^2^ = .022] for the 0 ms-centered pair. The Group x Task interactions were not significant for these two VOT pairs (both F<1). A significant group difference was only found for the +30 ms VOT pair [F(1,124) = 7.81, p < .01, η^2^ = .059]. There was also a trend for a Group x Task interaction for this pair [F(1,124) = 3.65, p = .058, η^2^ = .029]. Group contrasts on each task showed a significant difference on the observed d’ values [F(1,124) = 7.14, p < .01, η^2^ = .054], but not on the predicted d’ values [F(1,124) = 1.55, p = .215, η^2^ = .012]. That the difference between groups was larger with the discrimination task (i.e. for the observed values) than for the identification task (i.e. for the expected values) was expected on meta-analytic grounds [21]. However, the observed discrimination scores for the +30 ms VOT pair were larger for the CTL group than for the DYS group (Fig 1), suggesting that the DYS participants were *less* sensitive than the controls to differences around +30 ms VOT. This finding was unexpected insofar as the +30 ms VOT pair corresponds to a universal boundary [66] that is allophonic in French and DYS children have been reported to be *more* sensitive than controls to allophonic boundaries [33].

At this point, we are faced with two unexpected findings: (1) the absence of significant difference between groups for the 0 ms VOT discrimination score that is supposed to assess precision of the phonemic boundary and (2) the lower, instead of higher, discrimination by the dyslexic group for the +30 ms VOT pair that is supposed to reflect sensitivity to allophonic boundary. However, the above analyses rest on the assumption that the phonemic boundary is exactly located at the 0 ms VOT, although previous reports have shown that this boundary is in fact biased towards positive VOT values [63, 67]. The identification data collected in the present study confirms that the mean boundary is located above 0 ms VOT for both the DYS and CTL groups. They show that the distribution of the individual identification boundaries is skewed towards positive VOT values for the CTL group (Table 2). The asymmetrical distribution of the phonemic boundary towards positive VOT might have contributed to the unexpected results that were obtained here. A phonemic boundary that can take a range of values above 0 ms VOT might contribute to both the 0 ms and +30 ms VOT discrimination scores. Indeed in such a case, the 0 ms VOT score does not entirely capture phonemic discrimination and the +30 ms VOT score captures both phonemic and allophonic discrimination.

**Table 2:** Phonemic boundary: location along the VOT continuum and correlations with locations of the predicted and expected discrimination peaks.

Group	Phonemic boundary distribution		Correlation with location of the predicted peak	Correlation with location of the observed peak
	Mean (SD)	Skewness coefficient		
CTL	5.8 ms (13.1)**	-1.39 ***	R = .53 (p < .001)	R = .34 (p < .01)
DYS	4.0 ms (10.6)**	0.57	R = .65 (p < .001)	R = .14 (p = .29)

Departure of the mean boundary from 0 ms VOT, and skewness of the distribution

(***): p < .001

(**): p < .01.

To take account of the individual variations in the location of the phonemic boundary, further analyses were based on the discrimination peaks that were calculated separately for each participant and for each task. Both the amplitude of these peaks (in d’ values) and their locations along the VOT continuum (in ms) were taken into account.

The location of the “predicted” peak (calculated from the identification data) normally corresponds to the location of the phonemic boundary. Table 2 shows that the peak location indeed correlates fairly strongly with the boundary location for both groups. The amplitude of the predicted peak was therefore used to index the precision of the phonemic boundary. An ANCOVA with the location of the predicted peak as covariable showed a significant Group effect on Peak Amplitude [F(1,122) = 5.20, p < .05, η^2^ = .017] but no significant Peak Location effect [F(1,122) = 2.06, p = .15, η^2^ = .041] and no significant Peak Location x Group interaction [F<1]. DYS children showed a lower predicted discrimination peak, i.e. a lower precision of the phonemic boundary, a difference that did not depend on the location of the peak.

The location of the “observed” peak (calculated from the discrimination data) does not necessarily correspond to the location of the phonemic boundary (that is based on identification data). The observed peak location was found to correlate with the location of the phonemic boundary for the CTL group, but not for the DYS group (Table 2). The amplitude of this peak depends on both phonemic and allophonic sensitivity, in unknown proportions, and can thus not be used as an index of phonemic precision. However, as the allophonic boundaries are located at the endpoints of the VOT continuum, one might wonder whether the difference in observed peak amplitude depends on its location along the VOT continuum or not. An ANCOVA with the observed peak location as covariable showed a trend towards a Group effect on the Peak Amplitude [F(1,122) = 3.09, p = .08, η^2^ = .025] but no significant Peak Location effect and no significant Peak Location x Group interaction [both F<1]. DYS children thus showed a nearly significant lower observed discrimination peak than the controls, but this difference did not depend on the location of the peak.

In summary, examination of the predicted discrimination peaks showed that DYS children exhibit a lower precision of the phonemic boundary, independently of its location along the VOT continuum. Examination of the observed peaks also revealed a (nearly) significant difference between groups that was again independent of their location along the VOT continuum. This last result suggests that the enhanced observed discrimination skills of the controls are not specifically linked to allophonic boundaries.

#### Relationship between CP, phoneme awareness and VA span in the dyslexic population

Correlation analyses were computed for the whole dyslexic population between the precision of the phonemic boundary as CP index, phoneme awareness, VA span and reading scores. Boundary precision was assessed using the amplitude of the predicted discrimination peak, which is the sole CP index in the present study that presents the advantages to be clearly interpretable and to differ significantly between groups. Composite reading accuracy and reading speed scores were computed from the different word and pseudo-word lists (which correlated from 0.33 to 0.67 for accuracy and from 0.64 to 0.83 for reading speed). The phoneme awareness score corresponded to the mean accuracy percentage on the three phoneme awareness tasks (correlation coefficients from 0.33 to 0.43). The VA span score corresponded to the mean percentage of partial and global report tasks that correlated at 0.60. Partial correlations are presented on Table 3 after control of chronological age.

**Table 3:** Partial correlations (controlling for chronological age) for the whole dyslexic population (N = 63) between reading age, reading accuracy and reading speed, phoneme awareness, visual attention (VA) span and the amplitude of the predicted discrimination peak.

	Reading Age	Reading Accuracy	Reading Speed	VA Span	Phonological Awareness	Predicted Peak Amplitude
Reading age	1.000	.551***	-.684***	**.235~**	**.230**~	**.226**~
Reading Accuracy	.551***	1.000	-.441***	**.220~**	.275*	**.245**~
Reading Speed	-.684***	-.441***	1.000	-.312*	-.020	-.139
VA span	**.235**~	.220~	-.312*	1.000	-.060	-.100
Phonological Awareness	**.230**~	.275*	-.020	-.060	1.000	.252*
Predicted Peak Amplitude	**.226**~	**.245**~	-.139	-.100	.252*	1.000

In bold: significant and trend correlations

(***): p < .001

(*): p < .05

(~): .05<p < .09.

Results showed significant correlations between VA span and reading speed, and close-to-significance correlations between VA span and reading accuracy or reading age, while phoneme awareness correlated with reading accuracy (with a trend for reading age) but not with reading speed. Also in line with previous findings [3, 19, 20], no significant correlation was found between VA span and phoneme awareness. Interestingly, the precision of the phonemic boundary (as indexed by the amplitude of the discrimination peak) was not correlated with VA span abilities. Correlations between phonemic precision and both reading accuracy and reading age were close-to-significance. Critically, phoneme awareness skills correlated with phonemic precision.

#### Mediation analysis

**Fig 2:**
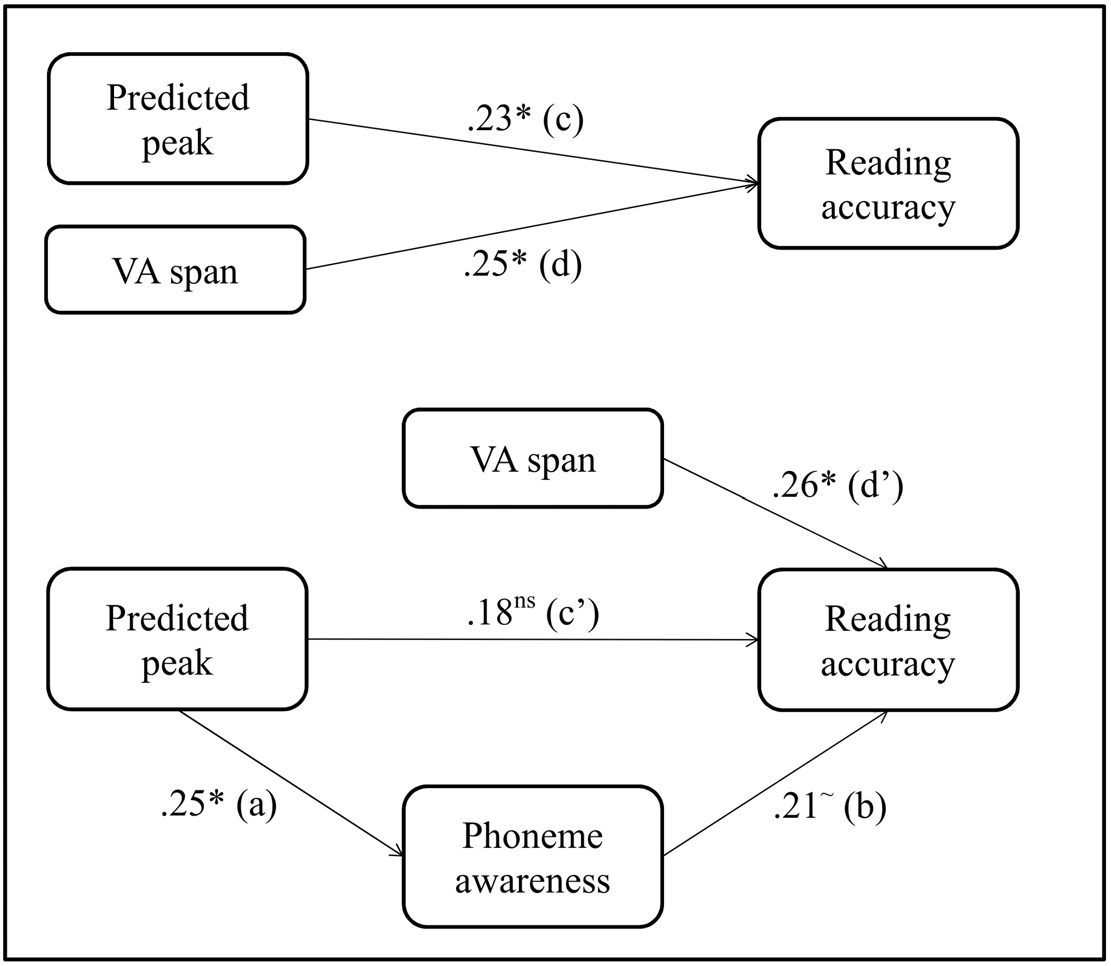
Schematic diagram of mediation analysis results. Path values are standardized regression coefficients. Significance levels are as follows: (*): p < .05, (~): p = .059, ns: non-significant.

We used mediation analysis [68] to test whether phoneme awareness mediated the effect of phonemic precision on reading accuracy. Mediation analysis allows exploring the relationship between an independent variable X and a dependent variable Y by explaining the mechanism by which X affects Y. In a mediation model, a third variable, the mediator variable M influences the effect of the independent variable X on the dependent variable Y. The effect of X on Y (path *c* in Fig 2) is referred to as the total effect. The effect of X on Y through M (paths *a* and *b* in Fig 2) is referred to as the mediated effect. The effect of X on Y in the mediated model (paths *c’* in Fig 2) is referred to as the direct effect. Several variables have been suggested to indicate a CP deficit in dyslexia. Various studies evidenced that dyslexic children exhibit a weaker precision of the phoneme boundary and/ or an enhanced sensitivity to acoustic differences within phoneme categories, that were either observed directly with discrimination data or derived from identification data with expected discrimination scores (see [21] for a review). Here, we adopted an exploratory procedure and selected the only variable for which a significant relationship with phoneme awareness was found. Significant relationships were only found when the precision of the phonemic boundary (as indexed by the amplitude of the predicted discrimination peak) was the independent variable and reading accuracy the dependent variable. The mediator variable for phonemic precision was phoneme awareness. A classic causal-step approach was conducted, i.e. a three-step multiple regression approach [69]. First, variations in phonemic precision should predict variations in phoneme awareness (path *a* in Fig 2). Second, variations in phonemic precision should predict variations in reading accuracy (path *c* in Fig 2). Third, phoneme awareness should predict variations in reading accuracy when phonemic precision is also included in the regression model (path *b* in Fig 2). The most important step to certify a mediated effect is to show that the effect of X on Y (path *c* in Fig 2) is no longer significant when the mediator variable is included in the regression model, i.e. phonemic precision should not predict reading accuracy when phonological awareness is taken into account (path *c’* in Fig 2). Because of the significant influence of VA span on reading accuracy, we also implemented this variable in the model (path *d* and *d’* in Fig 2) as an independent variable. To ensure that results from the mediation analysis were not influenced by a common effect of age, age was added as a regressor to all mediation regression models. The regression models used in the causal steps were thus the following:

Phoneme awareness=i1+j1Age+aPredicted peak+e1 (1)

Reading accuracy=i2+j2Age+cPredicted peak+dVA span+e2 (2)

Reading accuracy=i3+j3Age+c’Predicted peak+bPhoneme awareness+dVA span+e3 (3)

Results showed a significant influence of phonemic boundary precision on phoneme awareness skills and a close-to-significance (p = .059) influence of phoneme awareness on reading accuracy. The regression analysis further showed significant influence of phonemic precision and VA span on reading accuracy. But when phoneme awareness was included in the model, the direct influence of phonemic precision on reading accuracy was no longer significant, whereas the influence of VA span on reading remained significant. The indirect effect (a * b) calculated with Sobel test [70] was close-to-significance [Z-value = 1.40; p = .081].

## Part 2. CP in Cognitively Distinct Dyslexic Subgroups

Groups of dyslexic children with a single phoneme awareness disorder (PA DYS) or a single VA span disorder (VAS DYS) were identified to provide in-depth analysis of their CP abilities. CP of the two groups of dyslexic children was then compared to performance of the controls to explore whether the dyslexic group with a selective phoneme awareness deficit alone further exhibited a CP deficit (i.e., lower CP than the controls).

### Dyslexic subgroups

Results of the cognitive assessment were used to constitute groups of dyslexic children with single and distinct cognitive disorders. All dyslexic children whose Z-scores were inferior to -1.5 on at least one of the three phonological tasks (phoneme deletion, phoneme segmentation, or acronyms) were identified as phonologically impaired. The children with Z-scores below -1.5 on at least one of the report tasks (global or partial report) were considered as having a VA span disorder. Z-scores were computed for each school grade based on Bosse and Valdois [49]’s data for VA span and on the mean and standard deviation of the 63 controls of the current study for the phonological tasks. Seventeen dyslexic children were identified as having an isolated phoneme awareness deficit (27%), 20 showed an isolated VA span deficit (32%), seven children showed a double deficit (11%) and 19 none of these two deficits (30%).

The two groups with no deficit or the two deficits were not included in the analysis. The dyslexic children with both a phoneme awareness and VA span deficit were too few to be included. The dyslexic children belonging to the no-deficit group were not taken into account either, as we lacked evidence that they constituted a cognitively-homogeneous group. Some children of this group might have exhibited a cognitive disorder that differed from those taken into account in the current study. Others might be borderline on one or both of the PA or VA span tasks. Indeed, they had Z-scores superior to -1.5 on both the phoneme awareness and VA span tasks. So this subgroup includes both children with weaknesses in phoneme awareness and / or VA span (between -1 and -1.5), and children with within-the-average scores.

Comparisons between the two dyslexic groups with either a single phoneme awareness disorder (PA DYS; N = 17) or a single VA span disorder (VAS DYS, N = 20) were conducted through an ANCOVA with control of age (covariable) as the two dyslexic subgroups were not matched for chronological age [F(1,35) = 4.14; p = .05]. Performance of the two dyslexic subgroups on the cognitive assessment tasks is presented in Table 4. As shown on Table 4, the PA DYS and VAS DYS groups were matched for reading age and reading accuracy. The two groups significantly differed on reading speed, with slower reading speed in the VAS DYS group. Performance in phoneme awareness and VA span of the two PA DYS and VAS DYS groups is further provided to attest the two-groups’ double dissociation on these variables. Results are similar through an ANOVA when the VAS and PA subgroups are matched on chronological age, by eliminating the data of four VAS participants and one PA participant.

**Table 4:** Performance of the two dyslexic subgroups on the cognitive tasks and between-groups comparison.

Tasks	VAS DYS N = 20			PA DYS N = 17			Comparison VAS DYS vs. PA DYS	
	Mean (SD)	Range	Mean Z-score (SD)	Mean (SD)	Range	Mean Z-score (SD)	F (1,34)	*p*
Age (months)	120 (15.2)	94–143		130 (15.4)	105–153		covariable	
Reading age (months)	86 (5.5)	79–99		90 (4.9)	81–97		2.51	.122
Regular words Score (/20)	15 (3.5)	6–20	-2.90 (2.4)	16 (2.8)	12–19	-2.48 (2.2)	<1	.724
Regular words Time (second)	55 (18.7)	25–101	-3.74 (1.6)	37 (15.2)	21–86	-1.86 (2.2)	6.49	**.016**
Irregular words Score (/20)	10 (3.8)	4–18	-2.36 (1.4)	12 (4.1)	5–19	-1.77 (1.4)	<1	.768
Irregular words Time (second)	59 (19.9)	25–102	-2.89 (1.4)	44 (15.0)	21–77	-1.89 (1.9)	3.29	.079
Pseudo-words Score (/20)	11 (4.1)	4–18	-2.56 (1.8)	12 (3.3)	6–17	-2.44 (1.5)	<1	.940
Pseudo-words Time (second)	60 (18.4)	37–115	-2.42 (1.2)	45 (13.5)	27–79	-1.36 (1.5)	4.62	**.039**
VA Span								
Global report (%)	62 (7.4)	44–73	-1.94 (0.6)	76 (8.2)	59–94	-0.62 (0.7)	23.96	**< .001**
Partial report (%)	64 (12.6)	24–78	-2.24 (1.1)	82 (9.3)	66–96	-0.45 (0.8)	16.87	**< .001**
VAS composite score	63 (8.2)	34–71		79 (7.9)	66–94		30.75	**< .001**
Phoneme Awareness								
Phoneme deletion (%)	79 (12.6)	55–100	-0.19 (0.7)	56 (13.9)	35–80	-2.08 (1.1)	28.62	**< .001**
Phoneme segmentation (%)	63 (18.5)	27–100	0.08 (0.7)	43 (28.9)	7–93	-0.71 (1.1)	6.16	**.018**
Acronyms (%)	81 (11.2)	60–100	-0.05 (0.6)	52 (24.3)	0–80	-1.52 (1.3)	23.25	**< .001**
PA composite score	74 (10.7)	60–94		50 (12.6)	24–78		39.95	**< .001**

VAS DYS: Dyslexic children with a single visual attention span deficit. PA DYS: children with a single phoneme awareness deficit.

In the next sections, we have compared CP skills of each of the two cognitively distinct dyslexic groups with those of control children. For this purpose, each dyslexic group had to be matched with the control group on chronological age. The VAS DYS group matched the initial control group of 63 children on chronological age (F<1) (mean age = 10 years). As expected, they showed similar phoneme awareness skills as the controls [F(1,81) = 2.75; p = .101] but a lower reading age [F(1,81) = 100.64; p < .001]. However, the PA DYS group did not match the initial control group of 63 children on chronological age. Fifteen CTL children (the youngest ones) were then excluded and a new CTL group of 48 children was designed who had a similar chronological age as the dyslexic group (mean age PA DYS = 10 years and 10 months; mean age CTL = 10 years and 6 months. [F(1,63) = 2.44; p = .122]. As expected, the PA DYS showed a lower phoneme awareness performance than the controls [F(1,63) = 62.20; p < .001] and a lower reading age [F(1,63) = 93.80; p < .001]. Performance of each dyslexic group was then compared to each control group.

### Comparison of the PA DYS and CTL groups on CP skills

**Fig 3:**
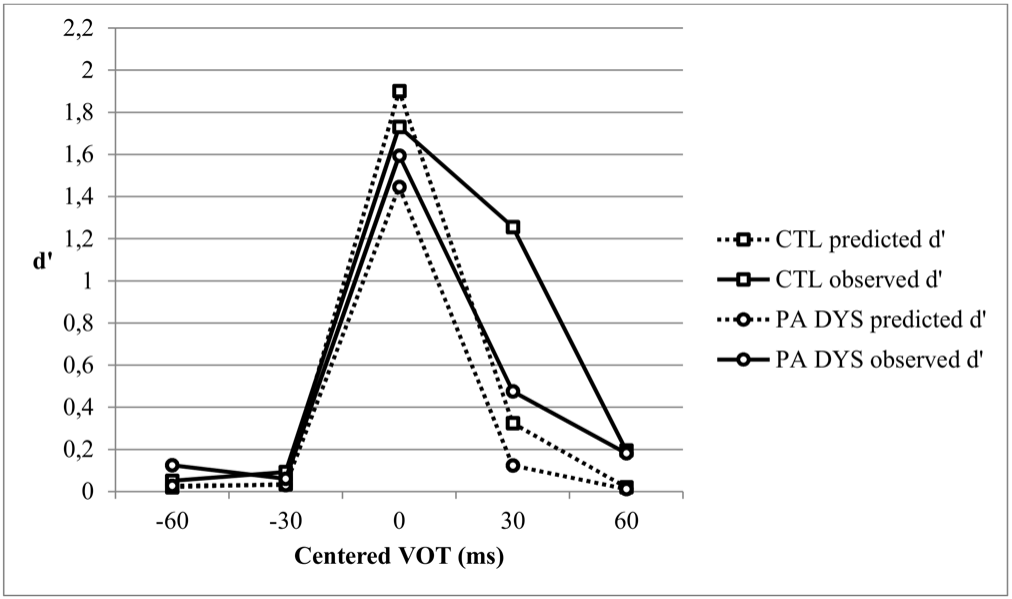
Predicted and observed discrimination curves for the Dyslexic subgroup with a single phoneme awareness deficit (PA DYS) and the Control Group (CTL).

Fig 3 shows the observed and predicted discrimination d’ scores for the age-matched PA DYS and CTL groups. A repeated measures ANOVA with Task (Observed vs. Predicted) and VOT (3 pairs centered on -30 ms, 0 ms, +30 ms, VOT) as within-participant variables, and Group (PA DYS vs. CTL) as between-participants variable, showed a significant Group effect [F(1,63) = 7.54, p < .01, η^2^ = .107]. The Group x VOT interaction [F(2,126) = 1.58, p = .215, Greenhouse-Geisser corrected, η^2^ = .024], and the Group x Task interaction [F<1] were not significant. However, there was a significant Group x VOT x Task interaction [F(2,126) = 3.60, p < .05, Greenhouse-Geisser corrected, η^2^ = .054], indicating that the Group x VOT interaction depended on the task.

An ANCOVA with the location of the predicted peak as covariable showed a significant Group effect on the amplitude of the peak [F(1,61) = 4.91, p < .05, η^2^ = .075] but no significant Peak Location effect [F(1,61) = 2.44, p = .12, η^2^ = .038] and no significant Peak Location x Group interaction (F<1). As for the whole dyslexic group, PA DYS children showed a lower predicted discrimination peak, i.e. a lower precision of the phonemic boundary, irrespective of the location of the peak. A similar ANCOVA conducted on the observed peak did not show any significant effects (all F<1).

### Comparison of the VAS DYS and control groups on CP skills

**Fig 4:**
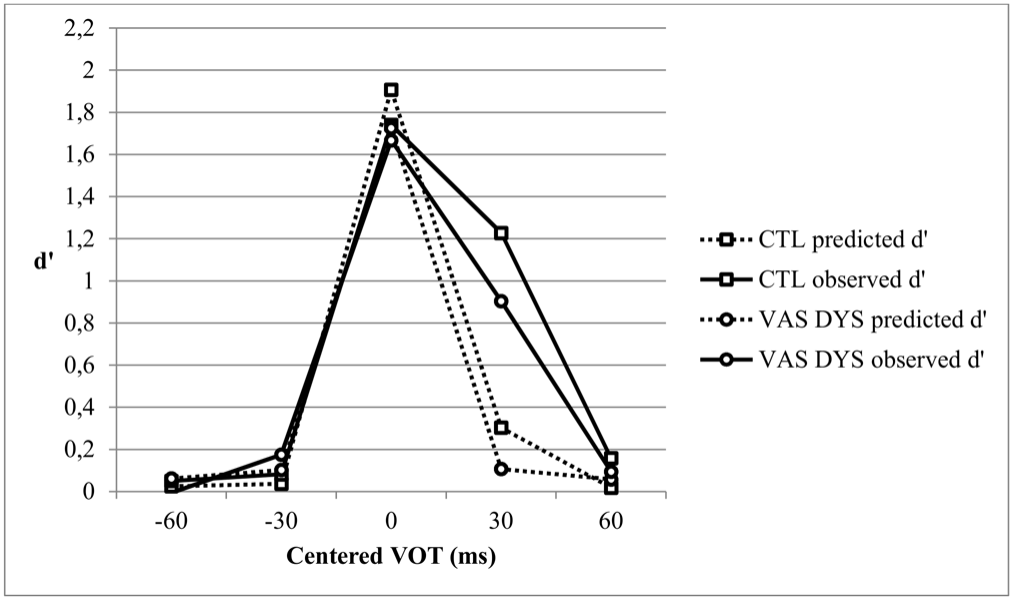
Predicted and observed discrimination curves for the Dyslexic subgroup with a single VA span deficit (VAS DYS) and the Control Group (CTL).

Fig 4 shows the observed and predicted d’ discrimination scores for the age-matched VAS DYS and CTL groups. As previously, a repeated measures ANOVA was conducted with Task (Observed vs. Predicted) and VOT (3 pairs centered on -30, 0, +30 VOT) as within-participant variables, and Group (VAS DYS vs. CTL) as between-participants variable. The Group effect was not significant [F(1,81) = 1.27, p = .263, η^2^ = .015]. The Group x VOT, Task x Group, and Task x VOT x Group interactions were not significant (all F<1), indicating that the Observed and Predicted discrimination curves did not differ between Groups.

An ANCOVA with the location of the predicted peak as covariable showed no significant between groups difference on peak amplitude [F(1,79) = 2.22, p = .14, η^2^ = .027]. There were no significant Peak Location effect and no significant Peak Location x Group interaction (both F<1). Thus contrary to the results obtained for the whole dyslexic group or the PA DYS group, the VAS DYS group did not exhibit lower phonemic precision than the controls. A similar ANCOVA conducted on the observed peak showed no significant effects (Group: F<1; Peak Location: F(1,79) = 2.46, p = .12, η^2^ = .030; Peak Location x Group interaction: F<1).

### Comparison of the VAS and PA dyslexic subgroups on CP skills

**Fig 5:**
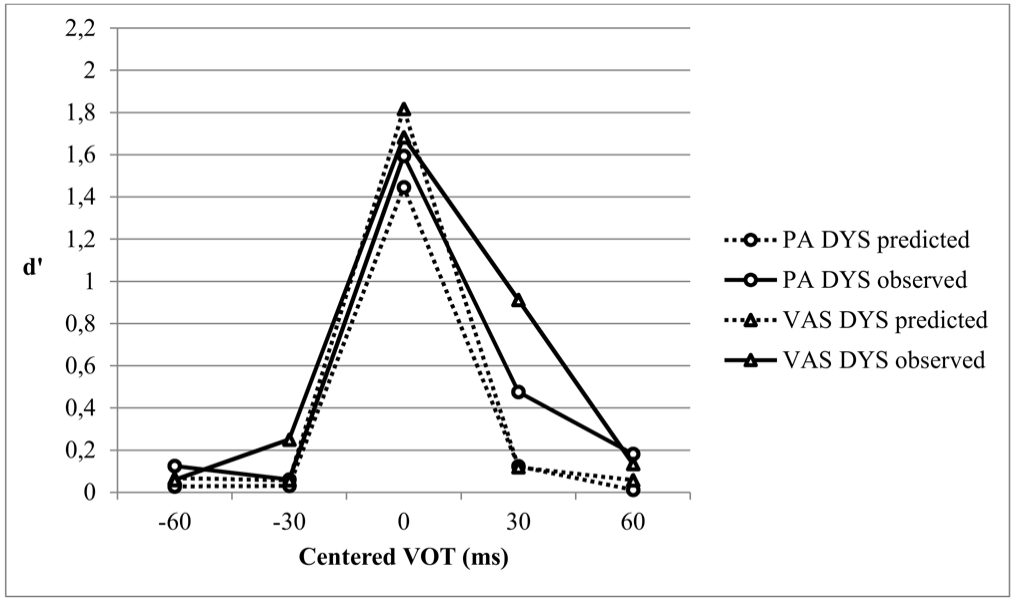
Predicted and observed discrimination curves for the Dyslexic subgroups with a single VA span deficit (VAS DYS) and a single phoneme awareness deficit (PA DYS).

To determine whether the selective CP deficit in the PA DYS group but not in the VAS DYS group further resulted in a significant CP difference between the two dyslexic groups, we matched the two groups on chronological age (thus excluding three VAS DYS children) and compared the new DYS groups’ CP skills. Fig 5 shows the observed and predicted d’ discrimination curves of these two DYS groups.

A repeated measures ANOVA was conducted with Task (Observed vs. Predicted) and VOT (3 pairs centered on -30, 0, +30 ms VOT) as within-participant variables, and Group (VAS DYS vs. PA DYS) as between-participants variable. The Group effect was not significant [F(1,32) = 2.42, p = .130, η^2^ = .070]. The Group x VOT, and Task x VOT x Group interactions were also not significant [F<1 and F(2,64) = 1.18, p = .303 Greenhouse-Geisser corrected, η^2^ = .036, respectively], indicating that the Observed and Predicted discrimination curves did not differ between Groups.

An ANCOVA with Peak Location as covariable showed no significant difference between groups in the amplitude of the predicted peak [F(1,30) = 1.16, p = .29, η^2^ = .037], no Peak Location effect [F(1,30) = 3.22, p = .08, η^2^ = .097] nor Peak Location x Group interaction (F<1). The VAS DYS and PA DYS subgroups did not differ in their phonemic precision. A similar ANCOVA conducted on the Observed Peak showed no significant effects (Group: F(1,79) = 1.16, p = .29, η^2^ = .037; Peak Location and Peak Location x Group interaction: both F<1).

## Discussion

In the current study, our main goal was to in depth explore the relationship between CP, phoneme awareness and reading. Our second purpose was to assess VA span skills in the same populations to establish the independence of VA span with respect to CP. The first part of the study was carried out on a large sample of dyslexic children who showed cognitive heterogeneity. The comparison of CP skills in the dyslexic and chronological-age-matched control children showed a CP deficit in the dyslexic population, characterized by lower precision of the phonemic boundary regardless of the boundary location.

Significant relationships were found between CP, phoneme awareness and reading accuracy. VA span correlated with none of the two former skills but did correlated with reading performance. Results of the mediation analysis suggested that CP modulates phoneme awareness skills, which in turn affect reading accuracy. The VA span independently affects reading accuracy, as this ability does not relate with either CP or phoneme awareness.

The second part of the study focused on two subgroups of dyslexic children taken from the initial population. A principal components analysis was performed to identify subgroups of dyslexic children with a single phoneme awareness or a single VA span disorder. The two subgroups were otherwise matched on reading age and reading accuracy. Their CP performance was compared to that of a chronological-age-matched control group. Results revealed that the group of dyslexic children with a single phoneme awareness deficit alone exhibited lower CP skills than the controls. CP skills were similar in the VA span impaired dyslexic children and control participants. However, direct comparison of the two dyslexic groups failed to show a significant difference in CP skills. The current findings provide new insights on the CP deficit in developmental dyslexia, on the relationship between CP, phoneme awareness and reading, and on the importance of considering the cognitive heterogeneity of the dyslexic population.

### The CP deficit in developmental dyslexia

As expected, dyslexic children were less sensitive than controls to differences across the phonemic VOT boundary [21]. However, these children did not exhibit an enhanced sensitivity to the -30 and +30 ms VOT allophonic universal boundaries. It is hardly surprising that ten year-old dyslexic children did not exhibit enhanced allophonic sensitivity. Behavioral evidence of allophonic sensitivity disappears with school experience in DYS children [71] but remains present at the neural level for both the DYS children and adults [72], suggesting an inhibition of neural sensitivity to the allophonic boundaries in the behavioral responses of DYS individuals.

More surprisingly, the dyslexic children in the present study were apparently less sensitive than controls to the +30 ms VOT allophonic boundary. It is worth noting that previous studies have consistently shown that typical French-speaking children and adults exhibit a discrimination peak in the positive VOT region [34, 63, 67] but that no study ever found enhanced sensitivity to the +30 ms VOT allophonic boundary in French children with dyslexia; the studies showed rather an enhanced sensitivity to negative VOT contrasts in dyslexic children [33, 34]. What is new with the present study is that children with dyslexia seem to exhibit a significant reduced sensitivity to positive VOT contrasts. A scrutiny of the current data revealed that such reduced sensitivity mainly resulted from interference with the phonemic boundary. Examination of between-group differences in the location and magnitude of individual discrimination peaks showed that the asymmetry in the distribution of the phonemic boundary was responsible for the apparently better allophonic sensitivity of the control group.

Despite the absence of allophonic sensitivity to negative VOT contrasts in the current results, a second marker of the CP deficit in dyslexia was significant, namely reduced precision of the phonemic VOT boundary. This was evidenced primarily by a lower magnitude of the predicted discrimination peak, that was located around the phonemic boundary for both the CTL and DYS groups. Another index of phonemic precision, the magnitude of the observed discrimination peak, was also lower for the DYS, but the difference between groups was only marginally significant. Moreover, this later variable was less easily interpretable because the location of the peak was not clearly related to the phonemic boundary for the DYS group.

To sum up, the present results evidenced a reduced precision of the phonemic boundary in the DYS group, which is the most common behavioral marker of the CP deficit in dyslexia. Another marker of the CP deficit in dyslexia, i.e. the enhanced sensitivity to allophonic boundaries, was not present here, presumably due to the fact that behavioral evidence of allophonic sensitivity disappears with school experience.

### Relationships between CP, phoneme awareness and VA span performance

Close-to-significance correlations were found between reading skills and the predicted discrimination peak and a significant correlation was found between the predicted peak and phoneme awareness skills which further correlate with reading skills. Mediation analyses were used to better grasp the links between CP, PA and reading in the dyslexic population. Results suggests that the relationship between CP, as indexed by the precision of the phonemic boundary, and reading accuracy may be mediated by phoneme awareness. Although the use of an exploratory procedure for the choice of the CP variable might inflate the risk of capitalizing on chance, our results are consistent with past-research findings that the direct relationship between CP and reading skills is typically subtle while the association of CP to phoneme awareness is stronger [39, 43, 45]. Using different phonemic contrasts and tasks of identification in typical children, McBride-Chang [46] also favored a model in which the effect of CP on reading was mediated by phoneme awareness. It nevertheless remains that the directionality of the relations among CP, phoneme awareness and reading is complex. On the one hand, empirical findings from longitudinal and training studies suggest that CP causally relates to phoneme awareness [47] and phoneme awareness to reading accuracy performance [1, 2, 73]. On the other hand, both phoneme awareness and CP skills increase with reading experience [71, 74] and better phoneme awareness may improve CP [74]. The overall findings thus suggest bidirectional associations, the complexity of which requires further investigations.

The current study for the first time investigated potential links between VA span and CP in developmental dyslexia. Previous evidence that CP relates to phoneme awareness while phoneme awareness and VA span are independent processes lead us to expect no significant relationship between VA span and CP, which was found. As previously reported in dyslexic [3, 19, 20] and typical readers [49], we found that VA span correlated with reading skills but did not relate to phoneme awareness. The clear relationship between CP and phoneme awareness in the absence of links between VA span and CP provides further support to the assumption of independence of the phoneme awareness and VA span disorders in developmental dyslexia.

The fact that phoneme awareness could mediate the relationship between CP and reading whereas VA span relates to reading but not to CP could explain some discrepant results reported in the literature [38, 75, 76]. Some of these discrepancies might follow from the heterogeneity of the dyslexic population. However, only a few studies have taken this heterogeneity into account [77], despite more and more evidence that different cognitive disorders characterize different subsets of dyslexic children.

Indeed, it is now well established that the VA span and phonological disorders typically dissociate in the dyslexic population ([3, 16, 19, 20], and see [55] for a neurobiological evidence) and that variability in VA span and PA skills independently influences reading performance in typical readers [48, 49, 78]. Recent findings further showed that poor VA span in developmental dyslexia is not just a consequence of the poor reading experience of dyslexic children [77]. To the contrary, there is now evidence that VA span causally relates to reading acquisition. Results from a recent training case study of developmental dyslexia showed that reading performance improved following an intensive and specific VA span training [18]. So, if the VA span disorder and the phonological deficit are two independent causes of developmental dyslexia, and if atypical CP specifically relates to poor phoneme awareness skills, then we would predict a CP deficit in phonologically-impaired dyslexic children but preserved CP in the VA span impaired DYS children who exhibit preserved phonological skills.

To explore this hypothesis, we zeroed-in on two groups of DYS children with a single phoneme awareness deficit (PA DYS) or a single VA span deficit (VAS DYS) and compared their CP performance to that of chronological-age-matched control children. Results showed that the PA DYS exhibited a CP deficit characterized by lower precision of the phonemic boundary. Conversely, CP was preserved in the VAS DYS participants who had no associated phonological disorder. The absence of CP deficit in this later group provides further evidence for a specific link between CP and phonological skills. It follows that all dyslexic children do not exhibit a CP deficit, which might account for the discrepant results previously reported in the literature [31, 38]. Preserved CP in VAS DYS but impaired CP in PA DYS further provide support for the existence of cognitively-distinct subtypes of dyslexic children. One subtype takes shape as characterized by phonological and CP deficits but preserved VA span while another subtype is characterized by poor VA span but preserved phonological and CP skills. Interestingly, previous studies further indicated that the first subtype alone showed poor sequential auditory processing [11, 79]. The absence of significant difference in CP between the two PA and VAS DYS subgroups was rather unexpected. We cannot rule out a relatively mild form of CP deficit in the VAS DYS group. However in such a case, the effect of this mild CP deficit on reading if any would not be mediated by phoneme awareness. Greater CP variability in the dyslexic subgroups might more likely account for the absence of direct evidence for a between-group CP difference.

## Conclusion

The current study suggests a special relationship between CP and phoneme awareness. A CP deficit, as indexed by a lower precision of the phonemic boundary is only found in the group of dyslexic individuals who show poor phoneme awareness. A second critical finding is evidence for the independence of the phoneme awareness/CP skills and VA span. These cognitive skills do not correlate, they independently contribute to reading accuracy and the VA span alone relates to reading speed. The overall findings suggest that a CP deficit might contribute to the phoneme awareness deficit in developmental dyslexia, independently of child’s VA span abilities. As intervention studies are the strongest tests of causal relations [23], remediation studies with programs targeting these specific cognitive deficits should play a major role in tackling this issue. Moreover, this approach, which takes into account the heterogeneity of the dyslexic population, may allow the development of more effective methods of cognitive remediation.
